# Safeguarding health equality for the disadvantaged during the COVID-19 pandemic: Lessons learned for the social work profession

**DOI:** 10.1177/1473325020973337

**Published:** 2021-03

**Authors:** Chi-Kin Kwan, Henry Wai-Hang Ling, Johnson Chun-Sing Cheung, Ernest Wing-Tak Chui

**Affiliations:** Department of Social and Behavioural Sciences, City 25809 University of Hong Kong, Kowloon, Hong Kong; Department of Social Work and Social Administration, The 25809 University of Hong Kong, Pokfulam, Hong Kong

**Keywords:** COVID-19, coronavirus, pandemic, epidemic, health equality, Hong Kong

## Abstract

An evaluation of the role played by the social work profession during the outbreak of COVID-19 is necessary. Although social workers have made efforts to address people’s needs during the pandemic, it is worth examining the role they have played in safeguarding health equality. Focusing on the case of Hong Kong, we found that the profession was generally ill-prepared for the outbreak, and in particular, for confronting the attendant social inequalities. We identified three possible reasons for these findings: 1) non-governmental organizations were caught off-guard by the outbreak, 2) there was no clearly articulated intervention agenda to inform practitioners of the roles they should play in such a large-scale crisis, and 3) having become more formalized and standardized, social work services may have become less flexible in responding to emerging community needs. We conclude this article by suggesting three directions that could allow the profession to better pursue its mission during large-scale crises.

Most people in the community have been negatively affected by the outbreak of COVID-19, but not equally so. During times of pandemics, poor populations are usually the most vulnerable (Ahmed et al., 2020). Although causal relationships have been found between large income differences and adverse health consequences (Pickett and Wilkinson, 2015), specific evidence on the health inequalities manifest during the COVID-19 pandemic is still emerging (Patel et al., 2020). As Giles (2009) notes, social workers need to develop a “health equality imagination” to deal with health inequality. Given that the profession is committed to promoting equality, social workers are expected to play an important role in confronting the inequalities that emerge during outbreaks of disease. However, many regular social work services did not operate during the recent pandemic, let alone address inequalities. The outbreak placed the social work profession in an awkward position and this is worthy of reflection. Focusing on the case of Hong Kong, we explore how the outbreak intensified already existing inequalities and led to greater suffering for disadvantaged people. We then reflect on the role that regular social work services have played during this unusual period and how our social work community can better protect the rights of disadvantaged people during times of crisis.

## Practicing social work during pandemics

Social work has been part of the health care system for more than a century (Judd and Sheffield, 2010). Accordingly, there is significant scope to explore the role the profession can play in tackling health inequalities, especially during a pandemic. The specific roles that social workers can play during pandemics can be traced back to the “Spanish Flu” in 1918 (Rosoff, 2008) and more recently to the SARS (Rowlands, 2007), H1N1 (Siu, 2012), and MERS pandemics (Park and Lee, 2016). However, no theoretical frameworks have been specifically developed to examine the roles that social workers play during pandemics (Chan et al., 2004; Cheung, 2020; Rowlands, 2007). In the health services sector, social work has long been influenced by the bio-medical model. Until recently, the global inequalities in health and well-being were largely ignored by the profession. As a result, the task of integrating health equality imagination and understanding into direct practice has been challenging for health social workers (Pockett and Beddoe, 2017). In addition to health social workers, community-based social workers need to make more concerted efforts to tackle health inequalities, especially those associated with the COVID-19 pandemic. Healy (2008) noted that social workers appear to place more emphasis on addressing human needs than human rights. This observation may be relevant when considering the roles social workers have played in the current pandemic.

## Safeguarding health equality during the COVID-19 outbreak

Social workers in Hong Kong have been shown to perform well in normal times, playing an important role in addressing various community needs (Chui et al., 2010). In addition to the more than 100 non-profit organizations that are funded by the government, several hundred non-governmental organizations (NGOs) provide a wide range of social services without regular government subvention. The establishment of the Hong Kong Social Workers Registration Board in 1998 provided mechanisms for monitoring their ethical standards. The Social Welfare Department also introduced the Service Performance Monitoring System to observe the service quality provided by the subvented NGOs (Kwan and Chui, 2018). Thus, it is reasonable to suggest that Hong Kong has a well-functioning social work sector that can deliver effective services to the community during normal times.

However, during the COVID-19 pandemic, Hong Kong’s social work community has been less effective in responding to the problems associated with the outbreak. The social work profession has not played an active role helping these groups fight for justice. With the current safety and crowd control requirements, the social work profession has only been able to provide essential and limited services. Many social workers were assigned to work from home in March and April, when the pandemic was at its peak. For instance, the day activity centers for people with disabilities and daycare centers for elderly people were temporarily closed. However, some of the service users had ongoing supervision needs. According to the observations of frontline social workers, the caregivers suffered greatly, with most of the daycare and face-to-face services (e.g., home visits and counseling) having been suspended.

COVID-19 has not only brought infection and death but also increased the vulnerability of the socially and economically deprived (Patel et al., 2020) and exposed the levels of digital inequality in society (Beaunoyer et al., 2020). Many Hongkongers acquired their own personal protective equipment (PPE) through various sources, including online retailers. However, PPE became both less affordable and less accessible for disadvantaged groups suffering from digital poverty. Similarly, when face-to-face classes were suspended, many children from low-income households lacked the resources to engage in digital learning at home. Other social problems, such as family violence and depression, appear to have become more serious during the outbreak. The “McRefugees,” or those who sleep in 24-hour McDonald’s restaurants, suddenly lost their shelters when the restaurants suspended their dine-in services. In light of this situation, it is worth examining whether the social work profession has responded effectively during the crisis.

## Our critical reflections

There are three possible reasons why social workers have not been able to effectively support disadvantaged groups during the pandemic or perform to their usual standards. First, NGOs appear to have been caught off-guard by the outbreak. As there was a need to help contain the spread of COVID-19, most service centers had to suspend their services. This was likely necessary, as many NGOs did not have sufficient PPE for their employees. Conflicts also occurred between frontline social workers and management over the resumption of services. Although many social workers could still work from home, they received inadequate ICT resources and training, and thus did not receive sufficient support to work remotely. For example, social workers require specific skills to provide effective online services. There are also issues of confidentiality when social workers contact their service users from home (Reamer, 2019), and these issues may not be covered by the regular guidelines.

Second, there is no clearly articulated intervention agenda to inform practitioners what role they should play in a large-scale crisis such as the COVID-19 pandemic. In particular, no intervention models are available to support practitioners in safeguarding the social rights of the disadvantaged. Although many social workers gain experience in dealing with crises at an individual level, many of the issues that have suddenly arisen during the current worldwide crisis, such as PPE allocation and digital poverty, are far more complicated and beyond the capability of the social work profession. In addition, although the large-scale challenges associated with the outbreak cannot be solved by any single profession, no effective inter-professional collaboration strategies have been developed to address these issues. Without a clear intervention agenda, most social workers can only respond to the crisis in a piecemeal manner.

Third, in recent years, social work services have become more formalized and standardized, and are thus less flexible in responding to sudden community needs. Formal support services play an important role in serving the needs of disadvantaged groups in Hong Kong. In this case, the service provision is primarily reliant on paid staff rather than the informal community networks. Nevertheless, during the peak of the outbreak, most services were suspended or could only provide limited services. Informal community networks may be comparatively more flexible in responding to immediate community needs. However, as Hong Kong mainly relies on professional-led services, the informal community networks tend not be sufficiently utilized. Coupled with weak community relationships, this has meant that the potential of the informal community networks has not been fully realized during the pandemic.

## Looking forward

The social work profession has learned a lesson from the COVID-19 outbreak. First, there is a need for social work agencies to develop contingency plans to enhance the capacity for social workers to continue to provide services during crises. Thus, the appropriate protocols and procedural guidelines need to be prepared. Social workers and service users should prepare for unusual and unforeseen events by conducting annual briefings and training. Social work agencies should also maintain inventories of PPE and other essential equipment to ensure normal service delivery during times of crisis and protect the safety of their staff. Moreover, NGOs should be prepared to deploy resources to support disadvantaged groups during crises. Secure and safe platforms for online counseling and computer systems for working from home should also be developed to ensure essential social work services continue to be provided during times of crisis such as pandemics.

Second, the social work profession needs to make sustained efforts to transform social justice from an aspiration into reality. In line with this objective, social work scholars and practitioners should work together to explore the roles that the profession should perform during times of crisis. Crisis intervention models should also be developed to inform practitioners of their roles during unusual times. As most of the issues arising from the outbreak cannot be resolved by each isolated profession, cross-sectoral collaboration should be further promoted and the profession should better utilize its expertise in liaising with stakeholders and coordinating resources. Strategies need to be developed to ensure that the voices of vulnerable groups can be better heard. To this end, there is a need for social workers to gain more political power to influence the policymakers.

Lastly, social workers should further promote the use and formation of community-based volunteer networks. It is a myth that community needs should be addressed by social services alone without utilizing the existing resources in the community (Ungar, 2004). Community-based volunteers can play a vital role in serving the needs of the community (Ling, 2018). It is also worth noting that informal volunteer service providers can be particularly helpful during emergencies and disasters (Whittaker et al., 2015). Ordinary residents, as trusted members of their communities, can provide timely support to other residents during crises. To better coordinate community resources, there is a need to explore the use of digital volunteer networks (Park and Johnston, 2017) in mobilizing and better coordinating the available community resources. For example, the online platform “Crowdtasking” (Neubauer et al., 2013), can help coordinate various community volunteers.

## Conclusion

The social work profession plays a crucial role in supporting people during times of crisis, especially disadvantaged groups (Giles, 2009; Pockett and Beddoe, 2017). As social justice is one of the core values of social work, the social work profession should make a concerted effort to address the inequalities that were highlighted during the outbreak of COVID-19. O’Leary and Tsui (2020) contend that we should not only be fighting the novel virus but also the injustice and discrimination that many innocent people have faced during the pandemic. Our mandate is to protect and connect those in the grassroots community by working together with other members of civic society with hope and love.
